# Age-related changes in pupil dynamics and task modulation across the healthy lifespan

**DOI:** 10.3389/fnins.2024.1445727

**Published:** 2024-11-19

**Authors:** Jeff Huang, Matthew L. Smorenburg, Rachel Yep, Heidi C. Riek, Olivia G. Calancie, Ryan H. Kirkpatrick, Donald C. Brien, Brian C. Coe, Chin-An Wang, Douglas P. Munoz

**Affiliations:** ^1^Centre for Neuroscience Studies, Queen’s University, Kingston, ON, Canada; ^2^School of Medicine, Queen’s University, Kingston, ON, Canada; ^3^Eye-Tracking Laboratory, Department of Anesthesiology, Shuang Ho Hospital, Taipei Medical University, New Taipei City, Taiwan; ^4^Department of Anesthesiology, School of Medicine, College of Medicine, Taipei Medical University, New Taipei City, Taiwan

**Keywords:** pupillary response, development, aging, saccade preparation, anti-saccade

## Abstract

The pupil is modulated by luminance, arousal, bottom-up sensory, and top-down cognitive signals, and has increasingly been used to assess these aspects of brain functioning in health and disease. However, changes in pupil dynamics across the lifespan have not been extensively examined, hindering our ability to fully utilize the pupil in probing these underlying neural processes in development and aging in healthy and clinical cohorts. Here, we examined pupil responses during the interleaved pro−/anti-saccade task (IPAST) in healthy participants across the lifespan (*n* = 567, 5–93 years of age). Based on the extracted measurements of pupil dynamics, we demonstrated age-related changes in pupil measures and task modulation. Moreover, we characterized the underlying factors and age-related effects in components of pupil responses that may be attributed to developmental and aging changes in the associated brain regions. Finally, correlations between factors of pupil dynamics and saccade behaviors revealed evidence of shared neural processes in the pupil and saccade control circuitries. Together, these results demonstrate changes in pupil dynamics as a result of development and aging, providing a baseline with which altered pupil responses due to neurological deficits at different ages can be studied.

## Introduction

The pupil is controlled by the balanced activity of the sympathetic and parasympathetic systems (Loewenfeld, 1999; Szabadi, 2012), and is modulated by luminance (Winn et al., 1994), arousal (Rajkowski et al., 1993), as well as both bottom-up sensory and top-down cognitive signals (Wang and Munoz, 2015; Joshi and Gold, 2020; Strauch et al., 2022). As it is a relatively low-cost and non-invasive measure, there has been growing interest in using pupil dynamics in various paradigms as a tool for probing the natural functioning and clinical dysfunction of these underlying processes. However, pupil physiology and neurological processes undergo dramatic changes across the lifespan. As such, it is essential to establish a normative trajectory of change in pupil dynamics across development and aging in order to fully utilize the pupil in studying different age and clinical groups.

Eye movement tasks have been valuable in the investigation of a wide range of sensory, motor, and cognitive functions. The pro−/anti-saccade task is well-established for investigating cognitive processing, requiring flexible executive control in order to generate correct eye movements according to task instructions (Munoz and Everling, 2004). Compared to stimulus-directed pro-saccades, anti-saccades involve looking in the opposite direction of a peripheral stimulus, which requires the suppression of stimulus-directed saccade signals and generation of new voluntary saccade signals on the opposite side of the brain. A large body of literature has employed this task to investigate changes in executive control in development and aging (Klein and Foerster, 2001; Luna et al., 2004; Munoz et al., 1998; Peltsch et al., 2011; Yep et al., 2022), as well as in various neurological disorders with executive control deficits (Amador et al., 2006; Boxer et al., 2012; Cameron et al., 2010; Chan et al., 2005; Green et al., 2007; Heuer et al., 2013; Kaufman et al., 2012; Munoz et al., 2003; Paolozza et al., 2013; Peltsch et al., 2008; Peltsch et al., 2014; Riek et al., 2023). These studies have demonstrated changes in saccadic performance related to developmental and aging processes as well as different neurological deficits.

Given the shared circuitry between pupil and saccade control, saccadic behavior and pupillary responses should show coordination (Wang and Munoz, 2021), and pupil dynamics should also exhibit age-related changes. Indeed, the preparation of a voluntary saccade is one of the cognitive processes modulating pupil size: in the interleaved pro−/anti-saccade task (IPAST), anti-saccades are associated with greater pupil dilation prior to peripheral stimulus onset compared to pro-saccades (Wang et al., 2015). Such a finding reflects previous neuroimaging studies that found stronger brain activation during the preparatory stage of anti-saccades compared to pro-saccades in frontal oculomotor regions (Connolly et al., 2002; DeSouza, 2003; Manoach et al., 2007) that have been shown to modulate pupil responses (Lehmann and Corneil, 2016; Ebitz and Moore, 2017). Furthermore, development and aging are associated with the maturation and degeneration of frontal cortical regions (Creasey and Rapoport, 1985; Casey et al., 1997; Luna et al., 2001; Bunge et al., 2002; Kramer et al., 2009) important to the age-related changes in anti-saccade performance (Coe and Munoz, 2017; Fischer et al., 1997; Klein and Foerster, 2001; Olincy et al., 1997; Peltsch et al., 2011), and frontal activations have been demonstrated to be strongly influenced by age (Alahyane et al., 2014). In Parkinson’s disease patients with executive control deficits, the modulation of pupil dilation by voluntary saccade preparation is disrupted (Wang et al., 2016), similar to the disrupted modulation in frontal oculomotor regions in this patient group (Cameron et al., 2012). Such evidence suggests that cognitive development and aging likely influence the pupil control circuit, and contributes to age-related changes in pupillary responses.

The goal of this study is to investigate age-related effects on pupil responses and the modulation of pupil size by saccade preparation in the IPAST. If components of pupil responses during saccade preparation reflect cognitive changes in natural development and aging, modulation of pupil dynamics by saccade task should vary as a function of age. Here, we examine changes in pupil dynamics across the human lifespan and investigate the link between pupil response and saccade behavior using the IPAST. We hypothesize that pupil dynamics are modulated by voluntary saccade preparation with age-related changes. We further characterize factors of pupil responses in the IPAST and examine their associations with closely linked saccade behaviors. With these findings, we consider the underlying neural substrates to explain pupil behaviors, and how maturation and deterioration in these areas may be responsible for age-related pupil changes.

## Materials and methods

### Participants

All experimental procedures were reviewed and approved by the Queen’s University Human Research Ethics Board (Ethics protocol File No 6005163; PHGY-007-97). Healthy participants with no known neurological or psychiatric conditions ranging between 5 and 93 years of age were recruited from the Greater Kingston Area, Ontario for a control cohort study (Yep et al., 2022). A total of 631 participants (409 females, 222 males) were included in this study, collected from April 2015 to February 2022. All participants self-reported no known visual, neurological, or psychiatric symptoms, had normal or corrected-to-normal vision, and were naïve regarding the purpose of the experiment.

A cognitive assessment was administered (Montreal Cognitive Assessment, MoCA) to all participants over the age of 18, and participants with MoCA scores below the cut-off of 20 were excluded from analysis. The cut-off score was determined based on MoCA score distribution from our adult participants. Compared to the standard cut-off of 26 (Nasreddine et al., 2005; Bourgeois-Marcotte et al., 2015), a lower cut-off was chosen as recommended by recent studies to lower the false positive rate for mild cognitive impairment in large, diverse samples including elderly adults (Rossetti et al., 2011; Carson et al., 2018).

Written informed consent was obtained from participants over the age of 18. Written informed assent, in addition to written informed consent from the parent or guardian, was obtained from participants under 18 years of age. Participants were compensated for their time in the study.

### Recording and apparatus

A video-based eye tracker (Eyelink-1000 binocular-arm, SR Research, Osgoode, Ontario, Canada) was used to measure eye position and pupil size with monocular recording of the right eye at a sampling rate of 500 Hz. Stimulus presentation and data acquisition were controlled by Eyelink Experiment Builder and Eyelink software. Visual stimuli were presented on a 17-inch LCD monitor at a screen resolution of 1,280 × 1,024 pixels (60 Hz refresh rate), subtending a viewing angle of 32° x 26°, and the distance from the eyes to the monitor was set at 60 cm, with a fixed head mount and chin rest to stabilize head position.

### Experimental paradigm

**Figure 1:**
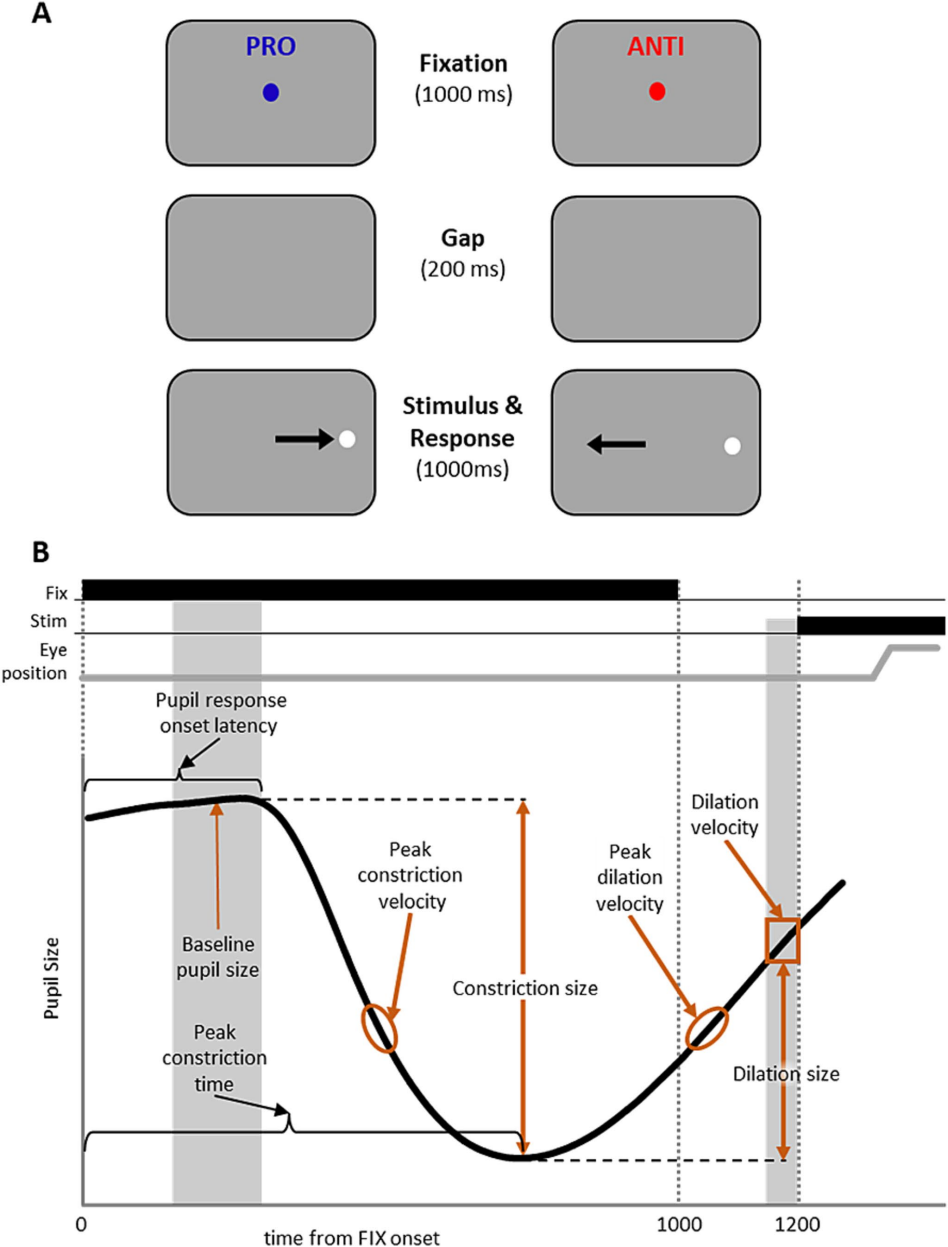
IPAST experiment paradigm and measurements of the pupil response. **(A)** Experiment paradigm for the interleaved pro- and anti-saccade task (IPAST). Each trial began with the appearance of a colored central fixation cue on a dark background. The color of FP provided the task instruction for the trial (Blue: PRO; pro-saccade. Red: ANTI; anti-saccade). After fixation, the FP was removed from the screen briefly, after which a white peripheral stimulus appeared horizontally to the left or right to the FP. Participants were instructed to either make a saccade to the stimulus location (PRO) or towards the opposite direction (ANTI). Note that the displayed arrows here indicating correct eye movement directions are for illustrative purposes only. **(B)** Measurements of the pupil response were calculated to capture the pupil dynamics, including baseline pupil size, pupil response onset latency, constriction size, peak constriction time, peak constriction velocity, dilation size, dilation velocity, and peak dilation velocity.

The experiment paradigm has been described previously (Yep et al., 2022; Coe et al., 2024). Participants were seated in a dark room, and the IPAST consisted of two blocks of 120 trials, lasting approximately 20 min. As illustrated in Figure 1A, each trial began with the appearance of a central fixation point (FP; 0.5° diameter, 44 cd/m^2^) for 1,000 ms on a black background (0.1 cd/m^2^). The color of the central fixation point provided the task instruction for the trial (green: pro-saccade; red: anti-saccade). Following 1,000 ms of fixation, the FP was removed from the screen, and the screen remained dark for 200 ms (gap period). After the gap period, a peripheral stimulus (0.5° diameter dot; gray, 62 cd/m^2^) appeared 10° horizontally to the left or right to the FP position. On pro-saccade trials (PRO), participants were instructed to make a saccade to the stimulus location as soon as it appeared. On anti-saccade trials (ANTI), participants were instructed to not look toward the stimulus, and instead look toward the opposite direction from the stimulus. The trial conditions (PRO and ANTI) as well as stimulus locations (left and right) were pseudo-randomly interleaved with equal frequency.

### Data analysis

Eye tracking data processing and general statistic analyses were performed using MATLAB (The MathWorks, Inc., Natick, MA, USA). Factor analyses were performed using SPSS version 28 (IBM, Armonk, NY, USA). Generalized additive model analyses were performed using R version 4.1.3 (2022).

First, eye-tracking data underwent pre-processing using a standardized pipeline (Coe et al., 2024), where data cleaning, saccade detection and classification, blink detection, and trial classification were performed to filter out data noise and categorize saccade and blink behavior at different periods during the trial. Saccadic reaction time (SRT) was defined as the time from stimulus appearance to the first saccade away from fixation. Trials where the first saccade after stimulus appearance were generated in the incorrect direction relative to the instruction were marked as direction errors, and were removed from subsequent pupil analysis. Accounting for the minimum 90 ms delay for afferent visual signals to trigger a saccade (Coe and Munoz, 2017), saccades were classified as anticipatory, express, or regular latency saccades based on their SRT (Coe et al., 2024). Anticipatory saccades, which are saccades made before subjects visually perceive the stimulus and indicate guessing behavior, occurred between −110 to 89 ms relative to stimulus appearance; express saccades occurred between 90 to 140 ms relative to stimulus appearance; regular latency saccades occurred between 140 to 800 ms relative to stimulus appearance.

We followed previous method (Steiner and Barry, 2011; Wang et al., 2012) to convert pupil size values recorded from the eye-tracker (in pixels) to actual pupil size (in mm). A series of different-sized false pupils (2 to 12 mm) were made and placed at the same position as participants’ pupil position during data recording. The recorded pupil values from false pupils were used to convert recorded pupil values from real participants to actual pupil diameter using a linear interpolation after taking the square root of the recorded pupil area data.

The pupil response profile in the IPAST has been consistently described (Wang et al., 2015, 2016; Perkins et al., 2021; Huang et al., 2023): the pupil initially constricted in response to the presentation of the fixation point, followed by pupil dilation that continued until stimulus appearance (Figure 1B). To capture the dynamics of the IPAST pupil response, we calculated 8 pupil measurements derived from the baseline, constriction, and dilation components of the response during the fixation epoch before stimulus appearance (Figure 1B). First, we removed transient noise in the pupil data by filtering high frequency pupil change (change in pupil size exceeded 0.1 mm/ms) and smoothing each data point with averaging ±25 sampling points. *Baseline pupil size* was calculated by averaging the pupil size during the epoch of 150 to 200 ms after FP onset, before the start of the pupil response. *Pupil response onset latency* was defined as the earliest point at which pupil velocity significantly differed from the baseline, calculated using a 20 ms sliding window. *Constriction size* was calculated as the difference between baseline pupil size and the pupil size at peak constriction, and the timing of this minimum pupil size was defined as the *peak constriction time*. *Peak constriction velocity* was calculated as the minimum velocity of pupil constriction during the fixation period prior to peak constriction time. *Dilation size* at the time of stimulus appearance was calculated as the difference between pupil size at peak constriction and the mean pupil size before target onset (−50 to 0 ms relative to target onset). *Pupil dilation velocity* at the time of stimulus appearance was calculated as the mean pupil velocity before target onset (−50 to 0 ms relative to target onset). *Peak dilation velocity* was calculated as the maximum velocity of pupil dilation in the fixation period after peak constriction time. Finally, the ANTI-effect for each pupil measure was quantified by calculating the difference between the median for PRO and ANTI trials in each participant (ANTI – PRO). Significant ANTI effect was determined using the Wilcoxon signed-rank test at *p* < 0.05.

Because pupil size is a sensitive measure and can be affected by blinks, noisy data, and eye position deviation, trials containing blinks or saccades (> 2° in amplitude) during the period of interest (from FP appearance to stimulus appearance) were excluded from analysis. Only trials with regular latency saccades (> 90 ms after target appearance) were included in the analysis in order to remove anticipatory saccades (Marino et al., 2012; Munoz et al., 1998). After these trial exclusion criteria were applied, each subject had to have a minimum of 10 correct PRO and ANTI trials for further analysis. Following these criteria, 64 participants (37 females, 27 males; 10.1% of total participants) were excluded from pupil analysis due to low MoCA scores or insufficient number of viable trials, and subsequent analyses were conducted with 567 participants (372 females, 195 males).

To investigate the relationship between pupil and saccadic behavior, correlation analysis was performed between the pupil measures of interest reported in the present study and the saccade measures collected from the same participants that have previously been reported (Yep et al., 2022). First, the 8 measures of pupil dynamics were each separated by PRO and ANTI conditions (with the exception of baseline pupil size and pupil response latency, which were combined across conditions due to lack of ANTI-effect), resulting in a total of 14 pupil measures. We performed factor analysis using principal axis factoring and oblique rotation (direct oblimin), considering the non-normally distributed data and for preserving accuracy for possible correlated factors (Costello and Osborne, 2005). Factor loadings at an absolute value >0.3 were considered significant given our sample size (Field, 2009). We further performed factor analysis on saccade behaviour measures repeating the procedures from Riek et al. (2023) using the same 12 saccade parameters. These include percentages of PRO and ANTI task disengagements, percentages of anticipatory pro-saccades and anti-saccades, mean PRO and ANTI SRT, percentage of express latency correct pro-saccades, percentages of express latency and regular latency ANTI direction errors, voluntary override time (VOT; Yep et al., 2022), and mean correct PRO velocity and amplitude. The four extracted saccade factors are subsequently labelled by the respective task-relevant brain processes they were theorized to measure in Riek et al. (2023): task disengagement (saccade factor 1), visual transient (saccade factor 2), frontal inhibition/voluntary saccade generation (saccade factor 3), and brainstem saccade generation (saccade factor 4). Similarly, we labelled the three pupil factors by the task-relevant brain processes they may represent and is further explained in the discussion: visual/luminance (pupil factor 1), top-down (pupil factor 2), and arousal/attention (pupil factor 3). Subsequently, Spearman’s correlation was performed to assess the relationship between the obtained pupil factors and saccade factors. Bonferroni correction was applied to adjust for multiple comparisons. Correlations were considered significant at *p* < 0.05.

**Figure 2:**
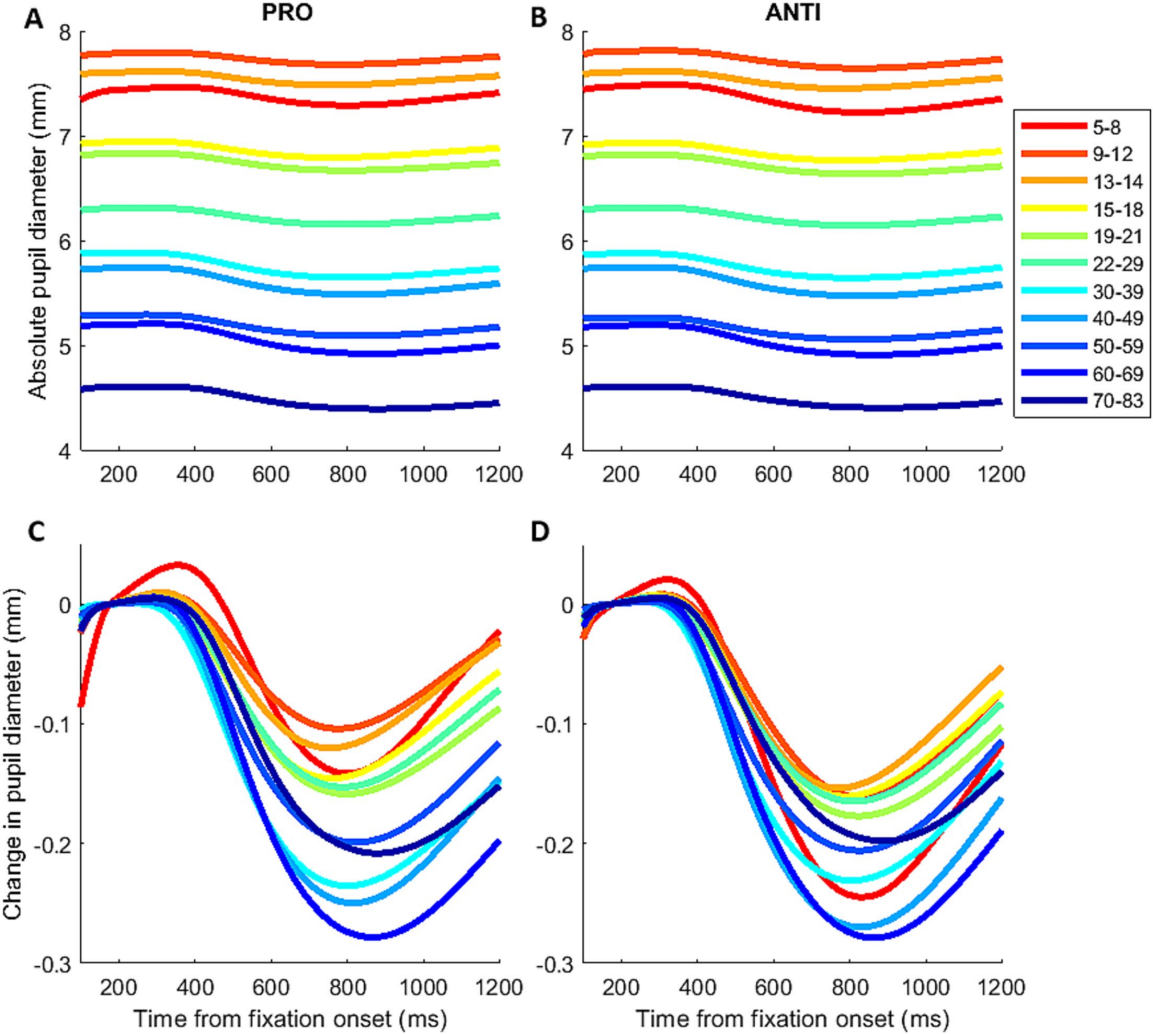
Mean pupil response across the lifespan. Mean pupil response before **(A,B)** and after baseline correction **(C,D)** for PRO and ANTI conditions during IPAST fixation period across the lifespan.

Many brain structures go through non-linear age-related changes over the course of the lifespan (Fjell and Walhovd, 2010). As the pupil control circuitry spans many cortical and subcortical areas, this likely results in a complex and non-linear age-trajectory of pupil dynamics. To qualitatively illustrate the changes in IPAST pupil response across individuals of discrete age groups, participants were divided into 11 age groups (Figure 2; Supplementary Figure S1). To assess the changes in pupil dynamics across the lifespan, pupil measures of interest were fitted using smoothing spline in MATLAB for qualitative examination, and following factor analyses the pupil factors were fitted with generalized additive models (GAMs; Hastie and Tibshirani, 1986) using the *mgcv* package in R (Wood, 2006). Analyses identifying significant periods of change were conducted using the *LNCDR* package in R, and significant periods of change were defined as the ages where the confidence intervals of the GAM fits’ first derivative did not contain zero (*p* < 0.05) (Tervo-Clemmens and Foran, 2022).

## Results

### Pupil dynamics in the pro- and anti-saccade task

Figures 2A,B illustrates the mean pupil responses for PRO and ANTI conditions from 11 subject age groups revealing the age effect on pupil baseline. To correct for baseline variations across individual trials, baseline pupil size was subtracted from pupil size values for each trial (Figures 2C,D). The overall pupil response profile appeared similarly as observed in previous IPAST studies, with an initial constriction, followed by dilation for participants of all ages (Wang et al., 2015, 2016; Perkins et al., 2021). Visual inspection did not reveal any obvious trends in the constriction component. On the other hand, the dilation component appeared to reduce in magnitude and velocity with increasing age. Furthermore, differences between the PRO and ANTI conditions displayed age-related trends (Supplementary Figure S1). In the youngest subject groups, the initial constrictions evoked by FP onset in ANTI trials showed smaller constriction magnitude, reached peak constriction earlier, and had larger and faster dilation compared to PRO. This pattern of differences between the two conditions appeared to reduce with increased age in both constriction and dilation components, as the two conditions show comparable pupil response in the older subject groups.

### Measurement of pupil baseline

**Figure 3:**
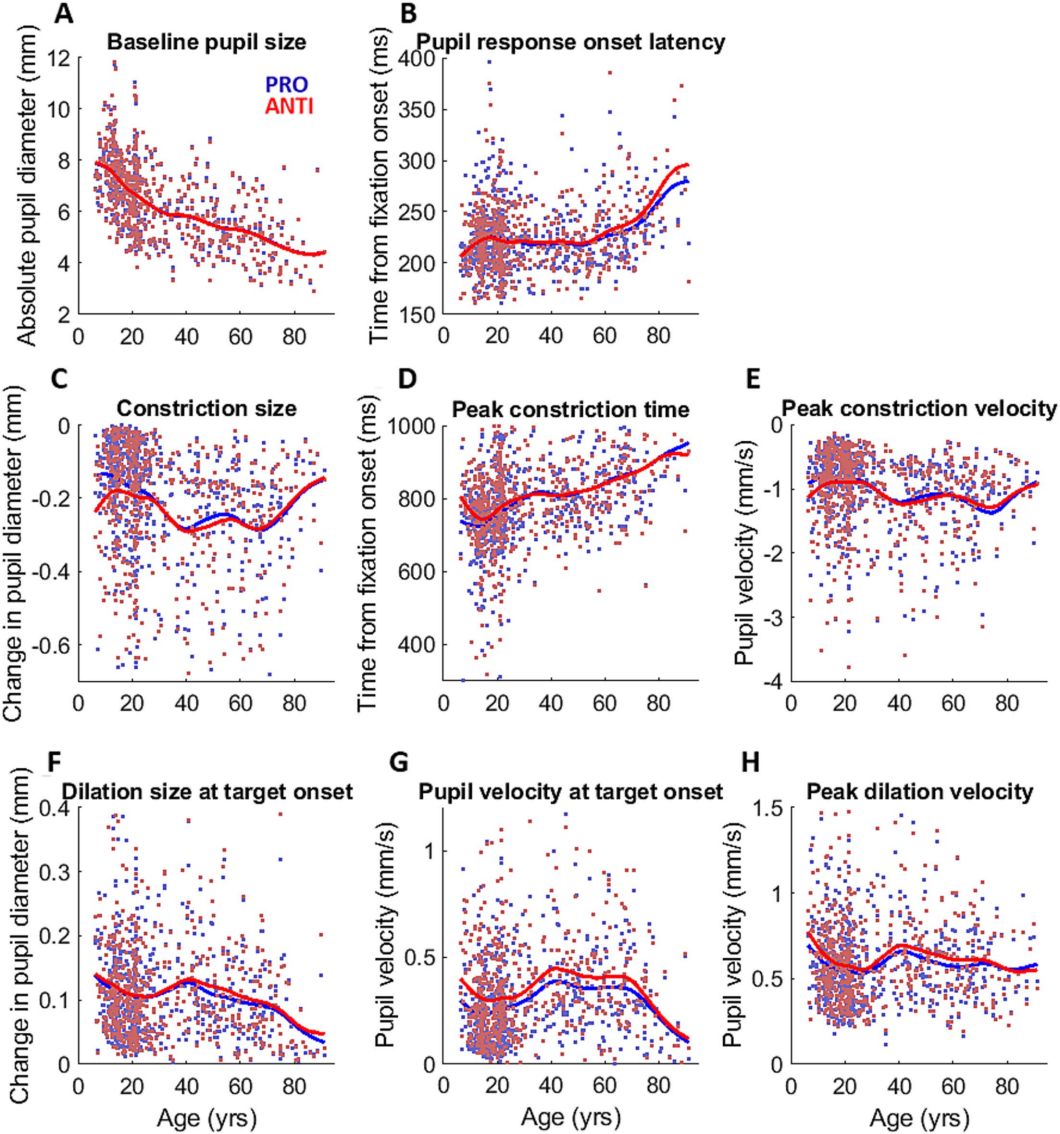
Pupil measurements across the lifespan. Measurements of pupil dynamics across the lifespan for PRO (blue) and ANTI (red) conditions: **(A)** baseline pupil size, **(B)** pupil response onset latency, **(C)** constriction size, **(D)** peak constriction time, **(E)** peak constriction velocity, **(F)** dilation size at target onset, **(G)** dilation velocity at target onset, and **(H)** peak dilation velocity. Colored dots represent individual subject data points, and colored curves represent smoothing spline fits of each task condition for all participants.

Figure 3A shows the changes in pupil size across the lifespan, as measured by pupil size at the start of each IPAST trial. Regardless of task condition, baseline pupil size decreased steadily during childhood and adolescence, followed by a more gradual decrease in adulthood. Additionally, we separated male and female participants to examine any possible sex differences on pupil size. A period of significant (*p* < 0.05) difference between male and female baseline pupil size was identified for the ages of 5–17.5, where female participants displayed significantly smaller pupil size compared to male participants (Supplementary Figure S2).

### Measurements of pupil dynamics

Figures 3B–H shows the changes across the lifespan of the extracted measurements of pupil dynamics during IPAST fixation. Pupil response onset latency (Figure 3B) remain largely unchanged until a gradual increase that began around 60 years. Constriction size and peak constriction velocity (Figures 3C,E) fluctuated across the lifespan but largely showed the constriction component becoming weaker as age increased, while peak constriction time (Figure 3D) displayed a steady increase over age. All three measures of pupil dilation displayed similar age trends (Figures 3F–H), showing a decrease from the youngest age to approximately 20 years, following by a small increase between 20 to 40 years, and then a general decrease after 40 years. Notably, dilation measures were higher for ANTI compared to PRO trials.

### Modulation of pupil dynamics by task condition

**Figure 4:**
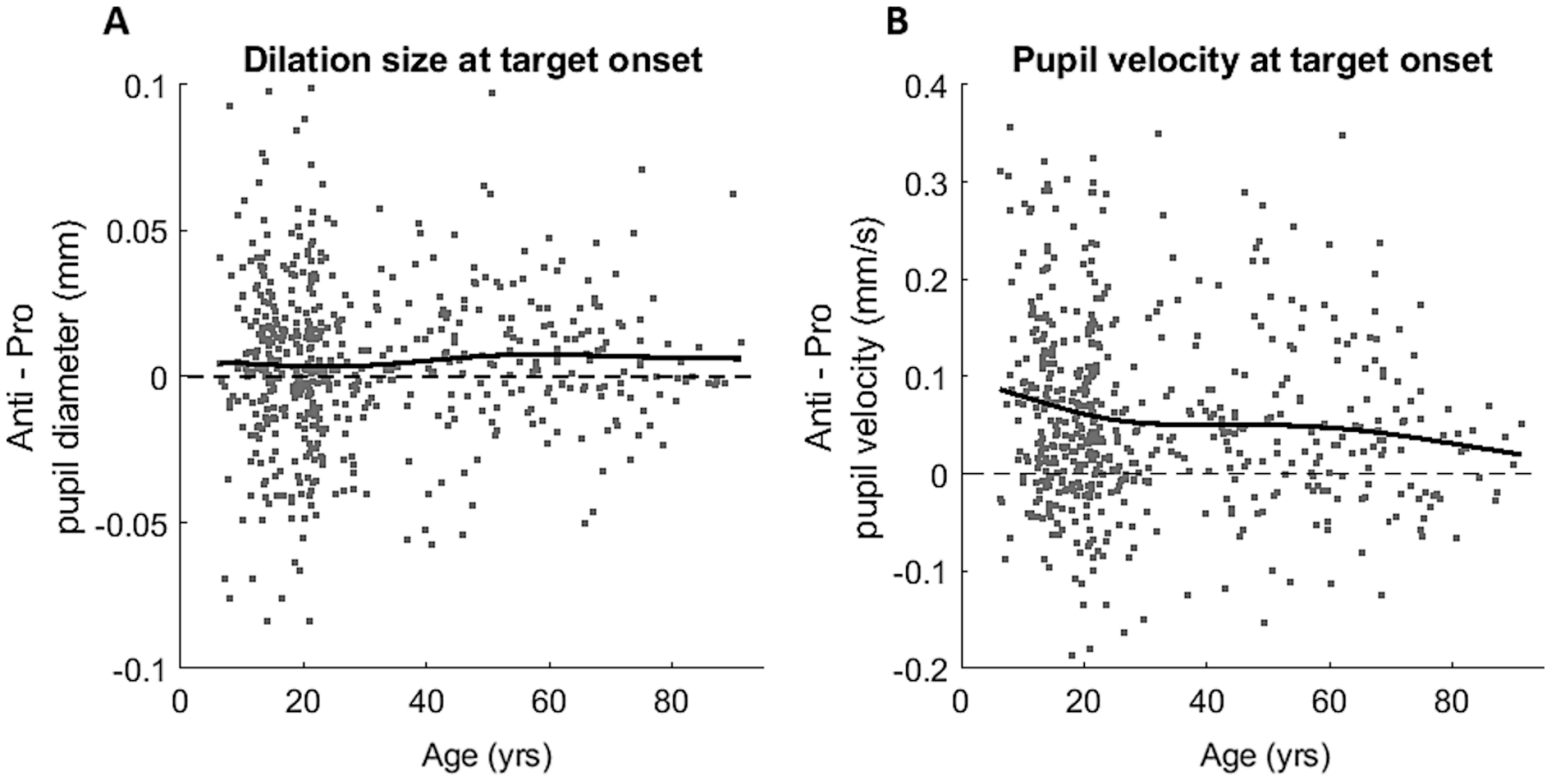
Task modulation (Anti-effect = ANTI – PRO) of the healthy lifespan for dilation size **(A)** and dilation velocity **(B)**. Grey dots represent Anti-effect calculated for individual subject, and black curves represent smoothing spline fits plotted for all participants.

To examine the effect of age on the ANTI-effect (the difference between PRO and ANTI conditions) of pupil response, we analyzed the relationship between age and the constriction and dilation measurements. Here we report four pupil measures that demonstrated significant ANTI-effect: constriction size (*p* < 0.001), peak constriction velocity (*p* < 0.001), dilation size (*p* < 0.001), and dilation velocity at stimulus appearance (*p* < 0.001). The ANTI-effect was quantified by calculating the difference between the subject averages of pupil measures for the two saccade conditions in each subject (ANTI minus PRO). For both constriction size and velocity (Supplementary Figure S3), the ANTI-effect weakened with age, where the weakening became less apparent after approximately 30 years of age. The ANTI-effect in dilation size, while present, was relatively maintained across the lifespan, whereas the ANTI-effect in pupil velocity at stimulus appearance weakened with age (Figure 4), mirroring the age trends of the constriction measures.

### Factors of pupil dynamics

**Figure 5:**
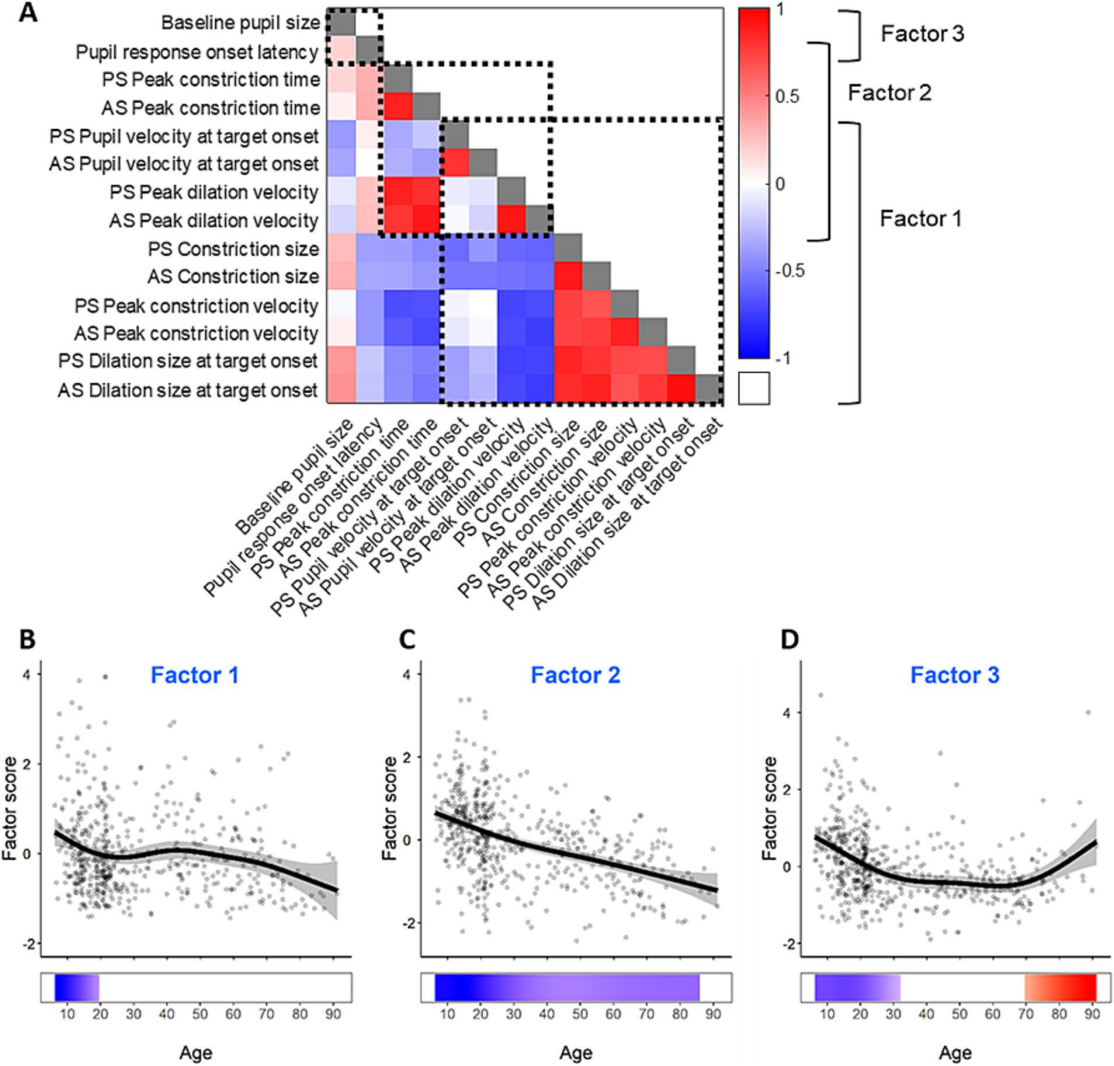
Factors of the measurements of pupil dynamics. **(A)** Correlation matrix and factor structure of the measurements of pupil dynamics. The color represents Spearman’s correlation coefficients between pairs of pupil measures; grey squares indicate correlation coefficients of 1. Dashed square outlines represent groups of pupil measures that loaded together in the factor analysis as displayed on the right. **(B–D)** Pupil factor scores for the three pupil factors across the lifespan. Grey dots represent individual subject factor scores, black curves represent GAM fits for all participants, gray ribbons are the 95% confidence intervals of the GAM fits, and the bottom tiles indicate periods of significant change.

Some of the pupil measurements demonstrated similar age trends, likely because of the continuous nature of pupil dynamics, and the overlapping signals driving components of the pupil response. To understand how these pupil measurements were related to each other and to provide insight into the underlying processes, we constructed an inter-individual correlation matrix using the 8 pupil measures for PRO and ANTI conditions (Figure 5A). We expected that measurements of the constriction component would be correlated, measurements of the dilation component would be correlated, and that there would also be correlations between constriction and dilation components due to the continuous nature of pupil response. Overall, there were reasonable significant correlations in the matrix consistent with this hypothesis.

To examine the relationships between the pupil measures, we further performed a factor analysis. The Kaiser-Meyer-Olkin measure demonstrated appropriate sampling adequacy (KMO = 0.785) and all KMO values for individual variables were above 0.5. Bartlett’s test of sphericity (*χ*^2^(91) = 11,781, *p* < 0.001) indicated sufficient correlations between variables for factor analysis. Three factors had eigenvalues above Kaiser’s criterion of 1 and explained 84.11% of the variance prior to rotation. Given the number of variables, large sample size, and average communality following extraction, > 0.7 Kaiser’s criterion was considered suitable, and three factors were extracted in the final solution. Constriction size, peak constriction velocity, dilation size, pupil velocity at stimulus appearance, and peak dilation velocity loaded onto factor 1; peak dilation velocity, dilation size, peak constriction time loaded onto factor 2; baseline pupil size and pupil response onset latency loaded onto factor 3 (Figure 5A).

GAMs were performed on the three pupil factors to examine their changes across the lifespan, and to identify periods of significant changes. The age effects are displayed in Figures 5B–D for the three factors. All three factors exhibited significant age-related changes. Factor 1 showed significant decrease from 5 to 20 years, but the gradual decrease after 40 years was not significant (Figure 5B). Factor 2 showed significant decrease across the lifespan at a relatively consistent rate. Factor 3 significantly decreased from 5 to 32 years, and significantly increased from 70 to 93 years.

### Relationship between pupil and saccade factors

**Figure 6:**
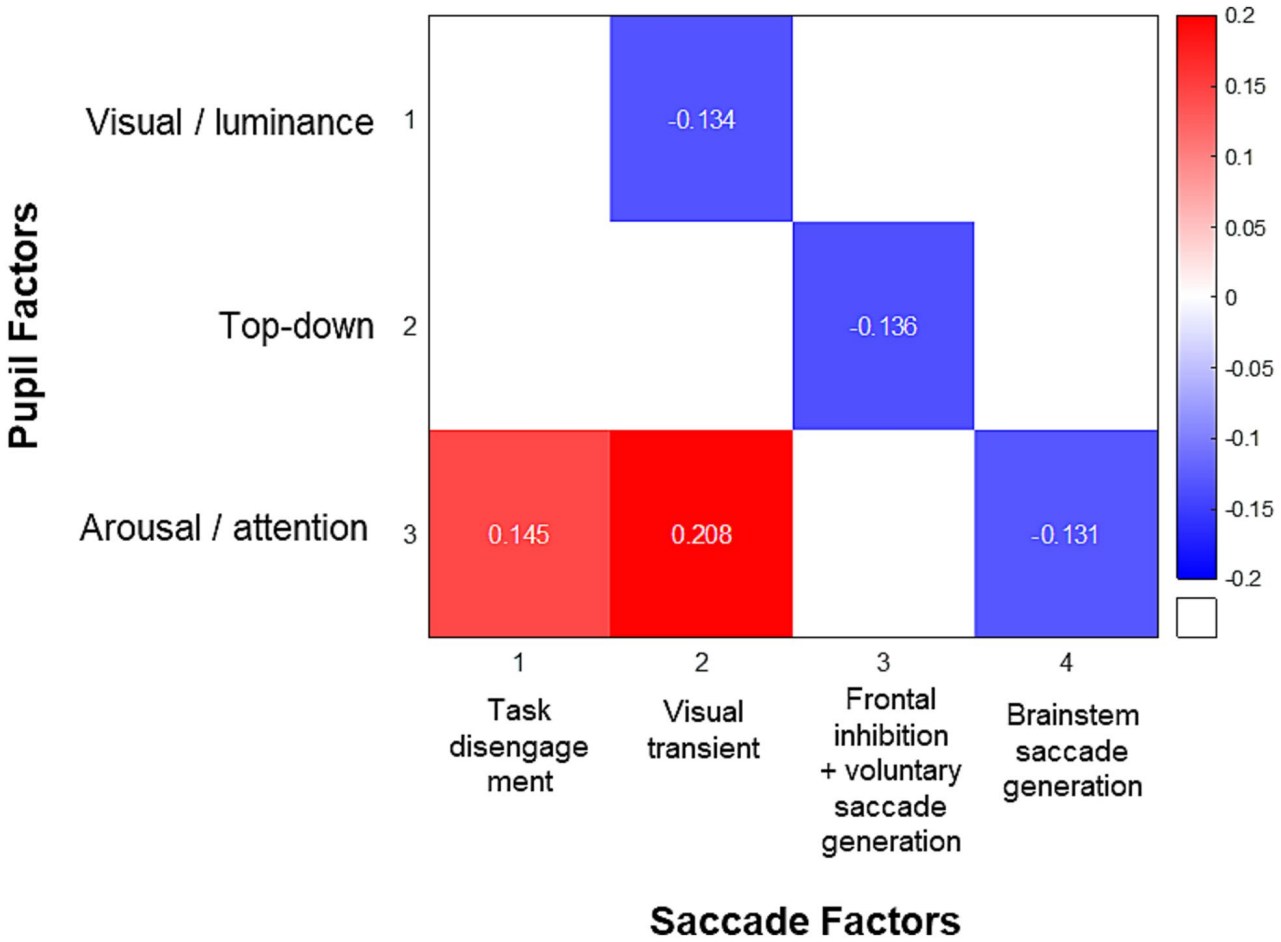
Correlation matrix of factor scores of pupil dynamics and saccade behaviors. The colored squares represent Spearman’s correlation coefficients between significantly correlated pairs of factor scores after Bonferroni correction.

Because of the overlapping circuitry underlying pupil and saccade control systems, pupil dynamics are associated with the preparation of voluntary saccades, and pupil dilation has been shown to correlate with SRT (Wang et al., 2015). The correlation matrix of saccade behavior measures in our healthy participants and the extracted saccade factors replicated the findings in neurodegenerative disease patients by Riek et al. (2023), and is displayed in Supplementary Figure S5. Importantly, to examine the relationship between pupil and saccade, and how the shared underlying processes drive both behaviors, we performed pairwise Spearman’s correlation between the factors of pupil dynamics and the factors of saccade behavior. Figure 6 illustrates the correlation matrix between pupil and saccade factors. Pupil factor 1 (visual/luminance) negatively correlated with saccade factor 2 (visual transient; *r* = −0.134, *p* < 0.005). Pupil factor 2 (top-down) negatively correlated with saccade factor 3 (frontal inhibition/voluntary saccade generation; *r* = −0.136, *p* < 0.005). Pupil factor 3 (arousal/attention) positively correlated with saccade factor 1 (task disengagement; *r* = 0.145, *p* < 0.001) and saccade factor 2 (visual transient; *r* = 0.208, *p* < 0.001), and negatively correlated with saccade factor 4 (brainstem saccade generation; *r* = −0.131, *p* < 0.005).

The saccadic performance findings of the same study participants have been reported in detail previously (Yep et al., 2022). As additional subject exclusion was done for pupil analysis for the present study (see Methods), to confirm behavioral validity, here we briefly reproduce two key saccadic performance measures in this subset of participants. Supplementary Figure S4A illustrates the mean SRT across age. Consistent with previous anti-saccade studies in healthy population across age (Fischer et al., 1997; Olincy et al., 1997; Munoz et al., 1998; Luna et al., 2004; Peltsch et al., 2011; Coe and Munoz, 2017; Yep et al., 2022), SRT of both pro-saccade and anti-saccades changed systematically with age. SRT reduced from the youngest participants to the early 20’s, followed by a steady increase with aging, and SRT was consistently lower for PRO compared to ANTI across the lifespan. Error rate also changed with age in a similar manner in the ANTI condition, while remaining consistently low across age in the PRO condition (Supplementary Figure S4B).

## Discussion

The goals of the present study were to characterize task-related changes in pupil dynamics across the healthy lifespan, and to investigate the relationship between pupil control and voluntary saccadic behavior in the IPAST. We hypothesized that pupil dynamics would be modulated by saccade preparation in the IPAST, and that such modulation would exhibit age-related changes. Our results demonstrated pupil modulation by saccade preparation in both constriction and dilation components of the response elicited in the IPAST. Importantly, modulations of these pupil dynamic parameters diminished as a function of age. Furthermore, we characterized the factors underlying pupil dynamics in the IPAST, and demonstrated the relationship between pupil and saccade factors. Together, our findings reveal changes in pupil dynamics across the healthy human lifespan and demonstrate that the pupil may be an effective probe for assessing development and aging changes in neural and cognitive processes in the human brain.

### Development, aging, and sex differences in pupil size

Pupil size is regulated by the opposing effects of two groups of smooth muscles: constrictor pupillae and dilator pupillae, which are innervated by the parasympathetic and sympathetic pathways, respectively. The pupil undergoes various changes across the lifespan, starting small in size from infancy and increasing during early childhood, after which pupil size gradually decreases with aging (Seitz, 1957; Kadlecova et al., 1958; Borgmann, 1972; Loewenfeld, 1979). Three factors have been postulated to be the reasons behind the small pupil size in childhood (Loewenfeld, 1999): (1) the smallness of the entire eye limiting the absolute pupillary size; (2) incomplete development of peripheral adrenergic activity; and (3) the immaturity of the brain and consequently low levels of mental and emotional activity as well as sympathetic discharges and central inhibition of the parasympathetic Edinger-Westphal nucleus. In a population survey which contained 1,470 participants from 1 to 100 years of age, Loewenfeld (1999) reported that the increase in pupil size during childhood continues until 11–12 years. More recently, Kohnen et al. (2004) reported a similar pupil size increase until the age of 11 years in 83 children between 1 and 14 years of age, while other studies with subject age ranges above 5 years did not observe this growth (Seitz, 1957; Borgmann, 1972). The lack of significant pupil size increase in the children in our study may simply be due to the smaller number of young participants in our study (e.g., *n* = 13 for 5 - 8 yrs), and the lack of any participants below 5 years of age. The difference in experimental conditions may also contribute to this difference, as these previous studies were performed with dark-adapted pupils with no task involved, compared to our active oculomotor task that also included a luminant fixation cue and peripheral stimulus.

Senile miosis is the well-known phenomenon of the natural reduction of pupil size in the eyes of the elderly (Kornzweig, 1954), but this decline in pupil size has been consistently reported to begin from much younger ages, soon after full pupil maturation has been reached in youth (Seitz, 1957; Kadlecova et al., 1958; Borgmann, 1972; Loewenfeld, 1979), as was shown in our findings (Figure 3A). This continuous decline is thought to be primarily caused by decreased central inhibition of the parasympathetic Edinger-Westphal nucleus with increased age (Loewenfeld, 1999). Since the smaller pupil size in childhood and aging can limit the range of pupil size, it may contribute to age-related reduction of pupil responses, and is therefore an important factor to consider.

Sex differences in pupil size and pupil response have been previously reported in various experimental conditions. However, there have been few findings published pertaining to the interaction between sex and age-related changes in the pupil. Sex differences during cognitive effort have also been previously investigated, however there have been mixed results. While some studies have reported female participants present with larger pupil than male participants during cognitive effort tasks (Campbell et al., 2018; Tsitsi et al., 2021), others have shown no pupil size difference between sexes during cognitive tasks (Toth and Campbell, 2019). We found pupil size was smaller in female participants than male participants between the ages of 5 to 17, which suggests developmental differences between the two sexes. A possible source of sex difference in the pupil in this age window is the locus coeruleus (LC), as age and sex differences have been reported in this structure (Clewett et al., 2016). As a key regulator of the pupil, the LC mediates arousal and has been widely attributed as the mechanism through which many aspects of cognition influence pupil size (Usher et al., 1999; Aston-Jones and Cohen, 2005; Gilzenrat et al., 2010). Further research is needed to shed light on the role of the LC in age-related sex differences in the pupil.

### Pupil dynamics in development and aging

One key brain area through which cortical signals converge to mediate pupil responses is the superior colliculus (SC), where top-down cognitive signals are integrated with bottom-up sensory signals to drive pupillary changes (Wang and Munoz, 2015). Pupil responses have been associated with various cognitive processes, including perception (Einhäuser et al., 2008), memory (Goldinger and Papesh, 2012), attention (Eldar et al., 2013; Naber et al., 2013), decision making (de Gee et al., 2014), and task preparation for saccades (Wang et al., 2015). Our results demonstrated that the modulation of pupil dynamics by saccade preparation changes in a non-linear pattern throughout the lifespan (Figure 4). Neurophysiological evidence has shown a direct link between pupil size and micro-stimulation in the frontal eye field (FEF; Lehmann and Corneil, 2016) and SC (Wang et al., 2012; Wang and Munoz, 2021), key areas for saccade preparation. Furthermore, FEF preparatory activation has been found to be reduced in children compared to adults (Alahyane et al., 2014). We therefore expected that the pupil modulation by saccade preparation should show a similar trajectory to saccade performance in the same task (Yep et al., 2022) begins weak in young children due to the delayed maturation of the frontal lobe, and reaches maximum in young adulthood when frontal maturation is complete, before slowly weakening due to age-related frontal deterioration.

We found that IPAST pupil dilation was strongest in the youngest participants, and decreased across the lifespan, with the most rapid decrease happening between 5 and 20 years of age, and after 70 years of age; the ANTI-effect of IPAST pupil dilation velocity also decreased across the lifespan. Several factors may have contributed to these findings. It is possible that the results with our youngest participants may be less reliable, as seen with the number of participants excluded for data quality and the high variability between young participants. On the other hand, this finding may instead provide insight for other brain regions underlying pupil control, as the relative contributions of different areas in the pupil control circuit may shift throughout development. The dorsal lateral prefrontal cortex (DLPFC) is one area linked to pupil dilation in cognitive tasks (Siegle et al., 2003) that may contribute to the larger pupil modulation in children. The DLPFC is a critical area for executive functions, and is part of the network of brain areas recruited in saccade preparation (Pierrot-Deseilligny et al., 2005). Indeed, while activations of most frontal oculomotor areas associated with saccade preparation increase with age throughout development (Luna et al., 2001; Hwang et al., 2010; Vink et al., 2014), literature has shown activation of DLPFC in saccade preparation to be of similar level between children and adults (Alahyane et al., 2014) or even decrease with age in other inhibition tasks (Casey et al., 1997; Durston et al., 2002; Velanova et al., 2008). A predominate influence of DLPFC on the pupil over other frontal oculomotor areas in children may very well explain our findings. This would suggest the possible involvement of the DLPFC through the SC (Johnston and Everling, 2006) in the preparation-driven pupil response particularly during development, but further research is required to shed light on the potential role of this area in modulating pupil dynamics.

### Neural correlates of pupil behavior in IPAST

Our factor analysis of the pupil measures produced three factors, which may represent distinct underlying neural processes driving IPAST pupil responses, and their age-related maturation and deterioration. The correlation analysis of pupil and saccade factors demonstrated associations for each of the pupil factors with one or more saccade factors, providing further insights into possible shared neural mechanisms between pupil and saccades.

Pupil factor 1 (visual/luminance) included measures of the constriction component, as well as dilation to a lesser degree, suggesting that it most likely related to the response to the visual stimulation of FP appearance. The significant period of change in pupil factor 1 occurred between 5 and 20 years of age, which encompasses the maturation periods of the different aspects of visual processing. For example, the maturation of visual acuity occurs between 5 to 15 years, and the maturation of contrast sensitivity occurs between 6 to 19 years of age (Ellemberg et al., 1999; Almoqbel et al., 2017). Pupil responses to visual stimulation have been well established as being modulated by various stimulus properties including luminance, color, and saliency (Gamlin et al., 1998; Loewenfeld, 1999; Oster et al., 2022; Wang et al., 2014). The visual-evoked pupil response could be conveyed by visual signals travelling directly via the retino-tectal pathway, and indirectly via the retino-geniculo-cortical pathway to the SC. The projection of SC to the pupil pathways then leads to the generation of pupil responses (Wang and Munoz, 2015). These pathways also provide visual input to the SC to guide visually-triggered saccades (Dorris et al., 1997). This is further supported by the observed correlation between pupil factor 1 (visual/luminance) and saccade factor 2 (visual transient), which was hypothesized to be driven by visual transient signals (Riek et al., 2023). However, given that visual function deteriorates in aging (Brewer and Barton, 2012), the decrease of pupil factor 1 in old age did not show significance, which may be due to the lower number of participants in our older age groups (e.g., *n* = 30 for 70 - 83 yrs).

Pupil measures that loaded onto pupil factor 2 (top-down) were those of the dilation component, suggesting that a neural process distinct from the visually evoked pupil response underlies the dilation component. Recent studies have associated pupil dilation with top-down cognitive control signals involving saccade preparatory processes through the SC (Wang et al., 2015; Wang and Munoz, 2018) and FEF (Lehmann and Corneil, 2016; Hsu et al., 2021). Pupil dilation associated with saccade preparation differs between pro- and anti-saccades (Wang et al., 2015). Pupil factor 2 decreases across the lifespan, likely related to the age-related changes in cognitive control in anti-saccades (Yep et al., 2022). The association of pupil factor 2 to voluntary saccade preparatory processes is further supported by its correlation with saccade factor 3, which was hypothesized to be driven by frontal inhibition and voluntary saccade generation, and correlated with multiple neuropsychological domains of cognition, including attention/working memory, executive function, and visuospatial function (Riek et al., 2023).

Tonic pupil size is associated with arousal (Bradshaw, 1967) mediated by the locus coeruleus-noradrenergic (LC-NA) neuromodulatory system (Rajkowski et al., 1993; Aston-Jones and Cohen, 2005; Murphy et al., 2014). Due to this link, pupil size has been frequently employed as a proxy measure for LC activity in studies of attention, fatigue, affective processing, and a wide variety of other processes where arousal is involved. Pupil factor 3, which baseline pupil size loaded onto, may be driven by the LC-NA system. The age-related change in this factor consists of a decrease before 32 years and increase after 70 years of age, which suggest that it may be an inverse of the LC-NA tonic activity, as LC signals have been shown to display an inverted-U pattern across the lifespan, reaching a peak around the age of 60 before rapidly decreasing (Manaye et al., 1995; Shibata et al., 2006; Clewett et al., 2016). Pupil factor 3 was correlated with three saccade factors: factor 1 (task disengagement), factor 2 (visual transient), and factor 4 (brainstem saccade generation). Indeed, LC-NA activity is thought to play a key role in regulating task engagement (Aston-Jones and Cohen, 2005), where LC activities in signal-detection tasks vary significantly in relation to false-alarm error rate (Kubiak et al., 1992; Aston-Jones et al., 1996; Usher et al., 1999), likely through modulating the ability to discriminate between target and distractors, and stimuli respond threshold (Aston-Jones et al., 1994). Furthermore, engagement in the form of “task preparedness,” where foveation prior to stimulus presentation was required in a task, was also shown to be associated with LC activity (Aston-Jones et al., 1996). LC-NA also plays a role in visual signal processing, enhancing visual signals at the levels of lateral geniculate nucleus (Campbell and Green, 1965; Rogawski and Aghajanian, 1980, 1982; Holdefer and Jacobs, 1994) and primary visual cortex (Kolta and Reader, 1989). Finally, the LC-NA system may be associated with brainstem saccade generation mechanisms through its projections to the oculomotor nuclei (Carpenter et al., 1992), and pharmacological evidence has shown that drugs modifying LC activity lead to changes in peak saccadic velocity (Haeusler, 1975; Aantaa, 1991; Glue et al., 1991; Coupland et al., 1994).

### Study history and design limitation

The present study began as an investigation of saccadic eye movement behaviors with a large cohort of healthy subjects across the lifespan (Yep et al., 2022), established in early 2014. The task design of the IPAST was optimized specifically for measures of saccade performance, and the value of the pupil data collected emerged afterwards. As a consequence, the study was not optimized for pupil data collection, and some aspects of pupil behaviors were overlooked. First, given the relatively long duration of a full pupil response and the IPAST trial duration of 3.2 s it is likely that one pupil response from one trial may not be fully complete within the trial duration, and may overlap with the subsequent trial, thereby contaminating our observed IPAST dynamics. Pupil recordings can also be disrupted by saccades and blinks during fixation, where the movements of the eye and the obstruction by the eyelids result in inaccurate or loss of pupil data. The increased blink rate during fixation in IPAST (Coe et al., 2024; Pitigoi et al., 2024), together with the elevated rate of direction errors for anti-saccades, which are particularly higher in the young and older age groups (Supplementary Figure S4B), can significantly limit the amount of viable data for analysis. Future studies targeting pupil as the measure of interest should control for these factors with care, and standards and recommendations in the field that provide important guidelines for pupillometry studies should be followed (Kelbsch et al., 2019; Steinhauer et al., 2022).

## Conclusion

Improvement in our understanding of pupillary control and how it is influenced by natural development and aging is essential, particularly given the growing interest in utilizing pupil measures to assess natural brain functioning and study various clinical disorders. Here we report changes in pupil response across the healthy lifespan, characterizing developmental and aging effects in aspects of pupil dynamics in relation to specific neural processes. These results establish a baseline with which abnormal pupil responses due to neurological deficits that may arise at vulnerable stages of development and aging can be studied. We further demonstrate correlations between distinct pupil and saccade factors, providing evidence for the associations between these eye behaviors and their shared utilities in probing various brain functions. Future work investigating longitudinal changes in pupil dynamics in healthy, neurodevelopmental, and neurodegenerative populations will provide important further insights into the underlying neural mechanism and elucidate the clinical utilities of pupil responses.

## Data Availability

The raw data supporting the conclusions of this article will be made available by the authors, without undue reservation.
