# Supplementary material for: Age-related changes in pupil dynamics and task modulation across the healthy lifespan
Source: Front Neurosci. 2024 Nov 19;18:1445727. doi: 10.3389/fnins.2024.1445727 (PMC11611812; doi:10.3389/fnins.2024.1445727)
Supplement: Supplementary file 1 [file Data_Sheet_1.DOCX]

## Supplementary Figures


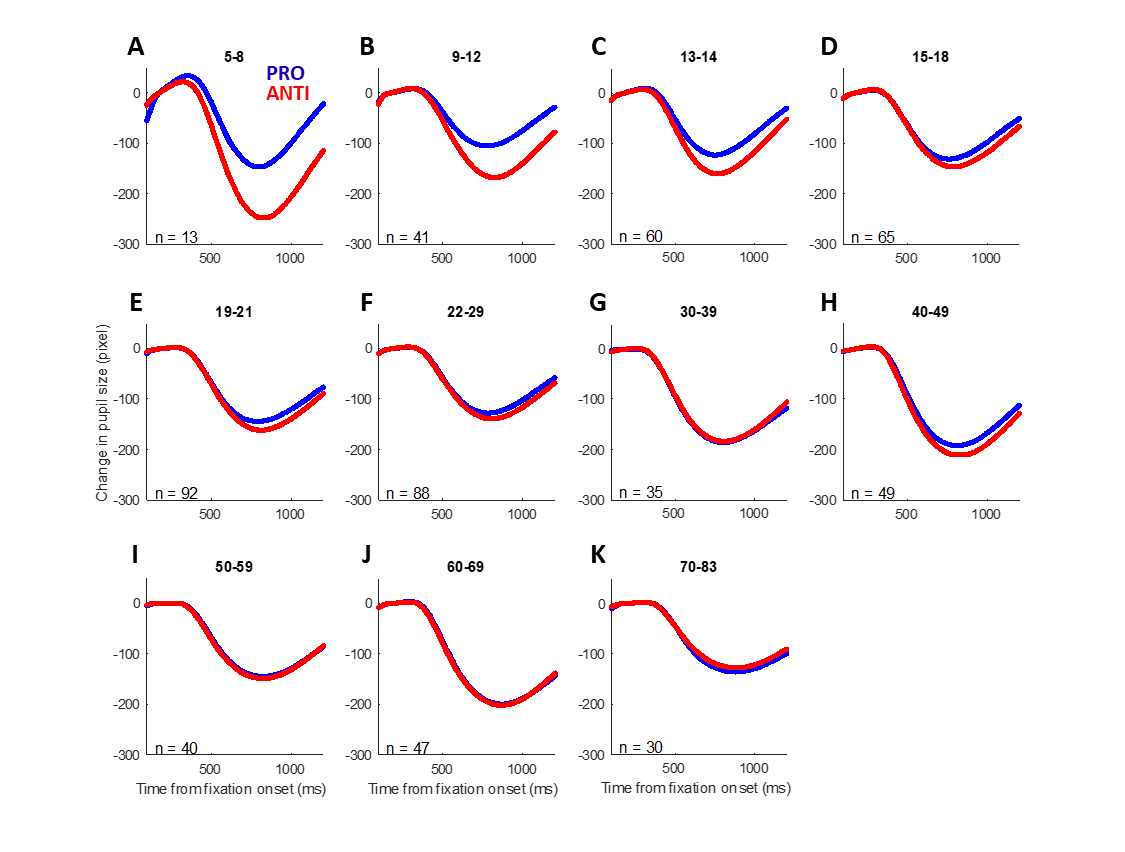


Supplementary Figure 1 Mean pupil response for PRO and ANTI conditions during fixation period across the lifespan

Age bins vary in size from 2-13 years, with smaller bins used before the age of 20 and larger bins used afterwards.


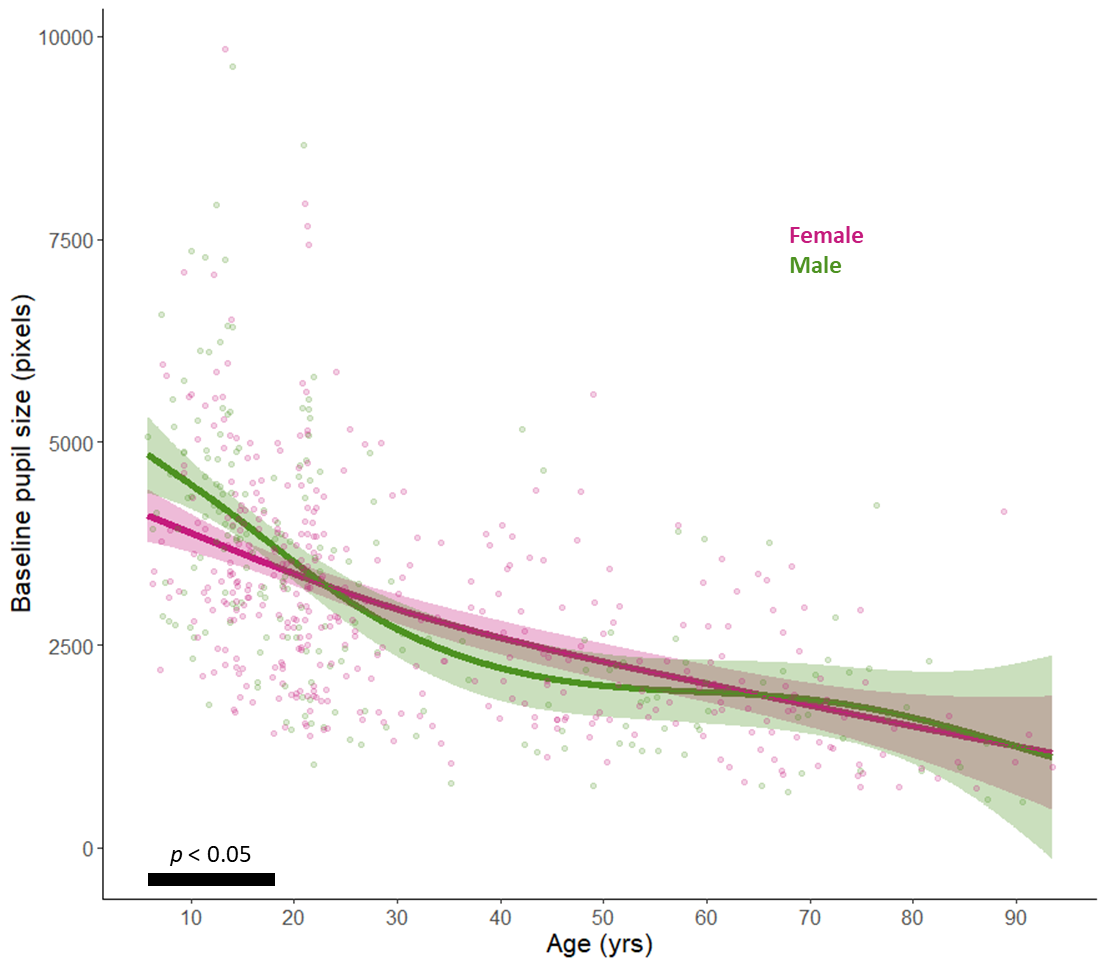


Supplementary Figure 2 Baseline pupil size in male and female subjects across the lifespan

Coloured dots represent individual subject data points, coloured curves represent GAM fits of male and female subjects across age, and shaded ribbons represent the 95% confidence intervals. Black bar indicate period of significant difference between two fits.

**
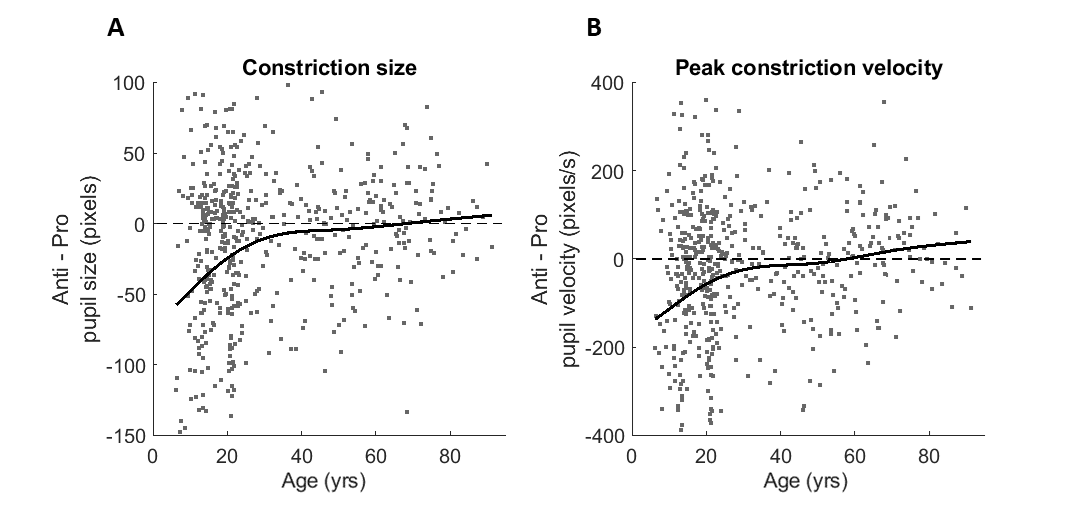
**

Supplementary Figure 3 Task modulation (ANTI-effect = ANTI – PRO) of pupil constriction across the healthy lifespan

Grey dots represent individual subject data points, and grey curves represent smoothing spline fits of the ANTI-effect for all subjects across age.


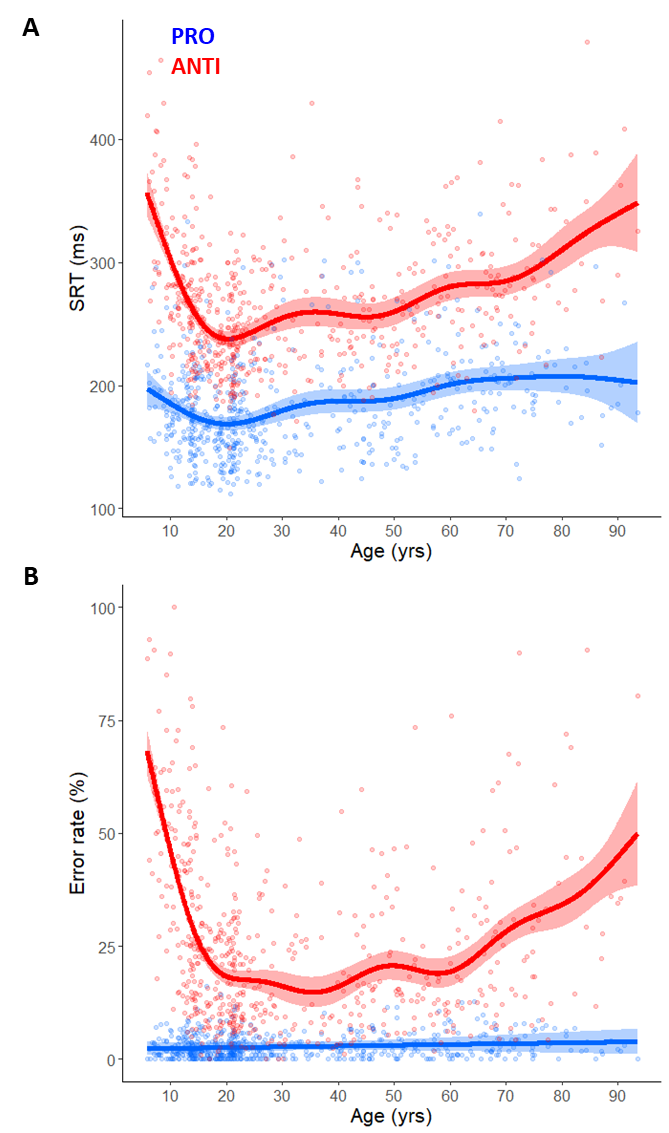


Supplementary Figure 4 Mean saccadic reaction time and error rate in the PRO and ANTI conditions across the lifespan

Coloured dots represent individual subject data points, coloured curves represent GAM fits of each task condition for all subjects, and shaded ribbons represent the 95% confidence intervals.


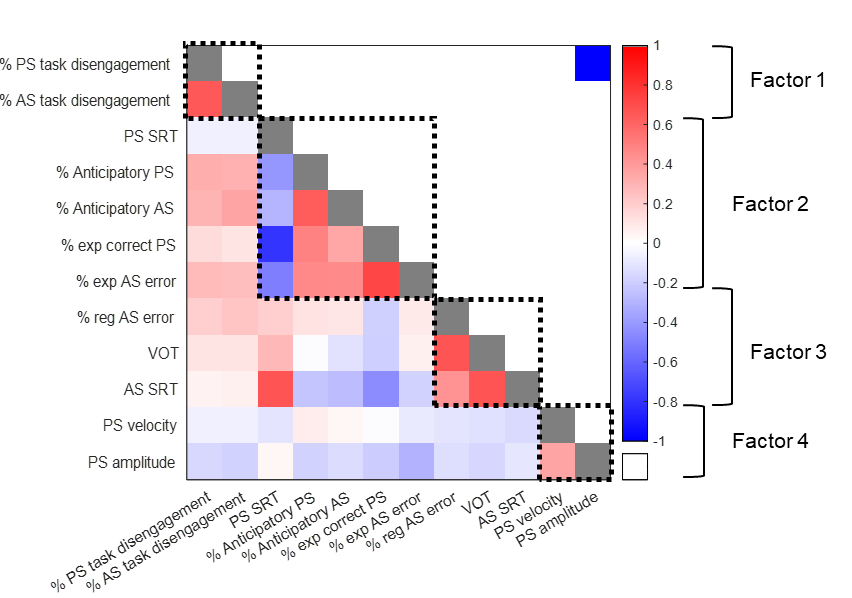


Supplementary Figure 5 Correlation matrix between measures of saccade behaviour

Colour bar indicates Pearson correlation coefficients between variables pairs; grey squares indicate correlation coefficients of 1. Black dashed boxes indicate groups of variables that loaded together on each factor. AS, antisaccade; PS, prosaccade; SRT, saccadic reaction time; VOT, voluntary override time.
