# Supplementary figures and images for: Age-related changes in pupil dynamics and task modulation across the healthy lifespan
Source: Front Neurosci. 2024 Nov 19;18:1445727. doi: 10.3389/fnins.2024.1445727 (PMC11611812; doi:10.3389/fnins.2024.1445727)

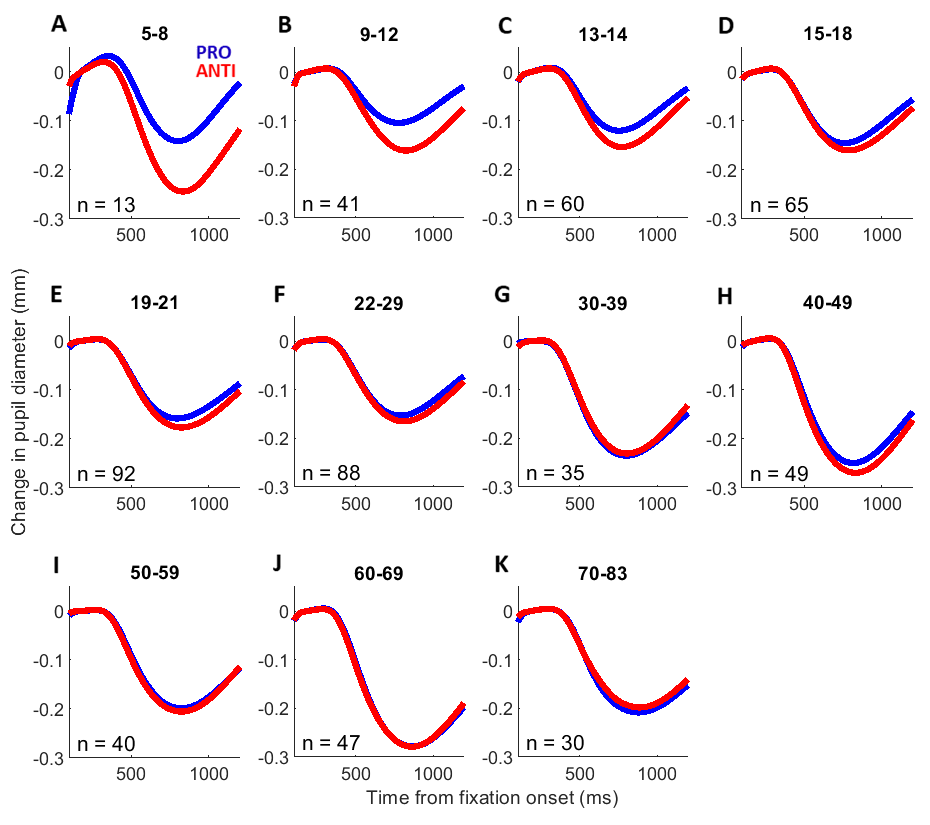

Supplement: Supplementary file 2 [file Image_1.TIF]

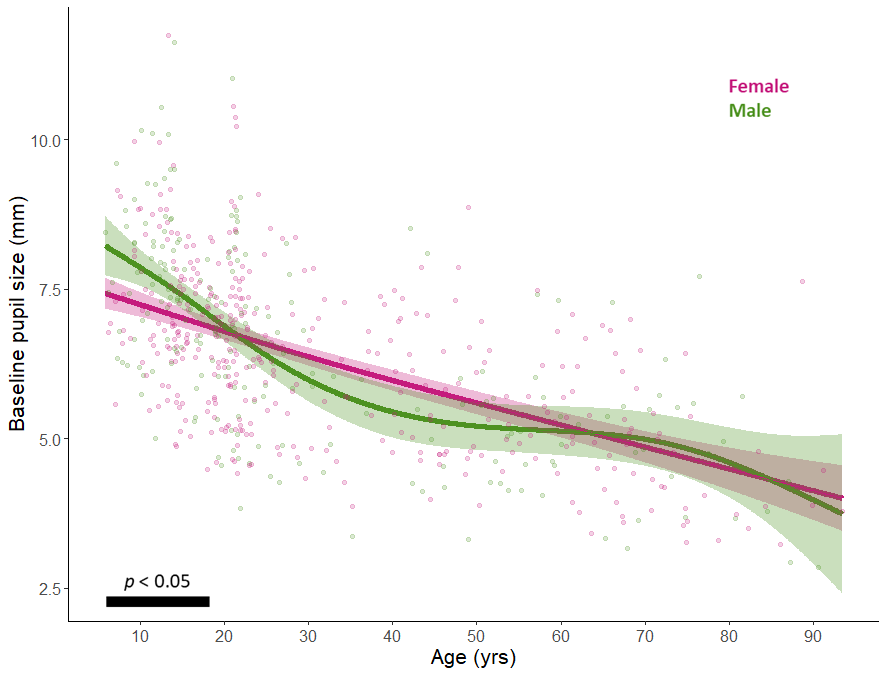

Supplement: Supplementary file 3 [file Image_2.TIFF]

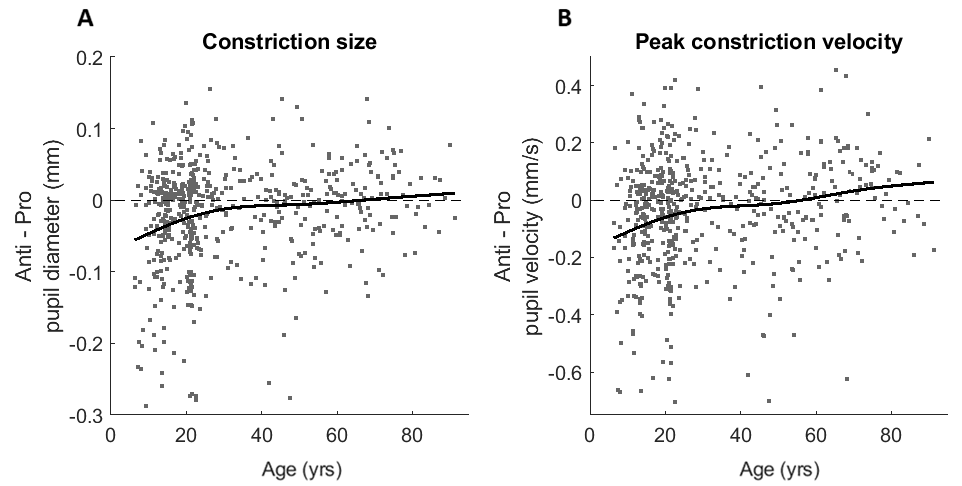

Supplement: Supplementary file 4 [file Image_3.TIF]

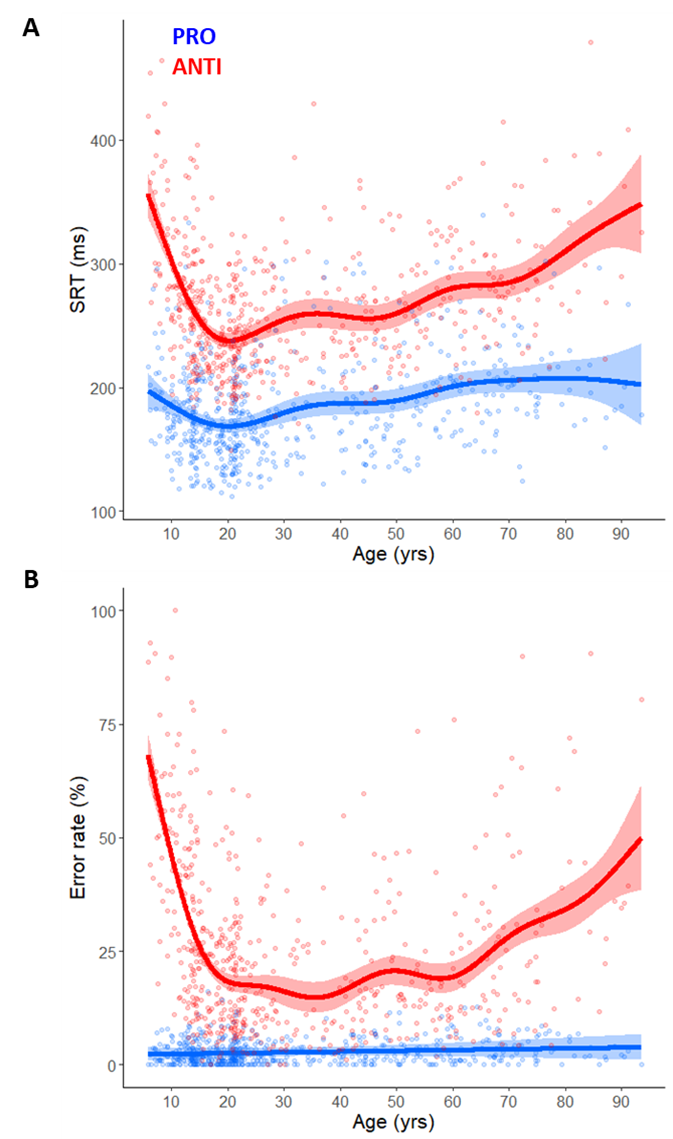

Supplement: Supplementary file 5 [file Image_4.TIF]

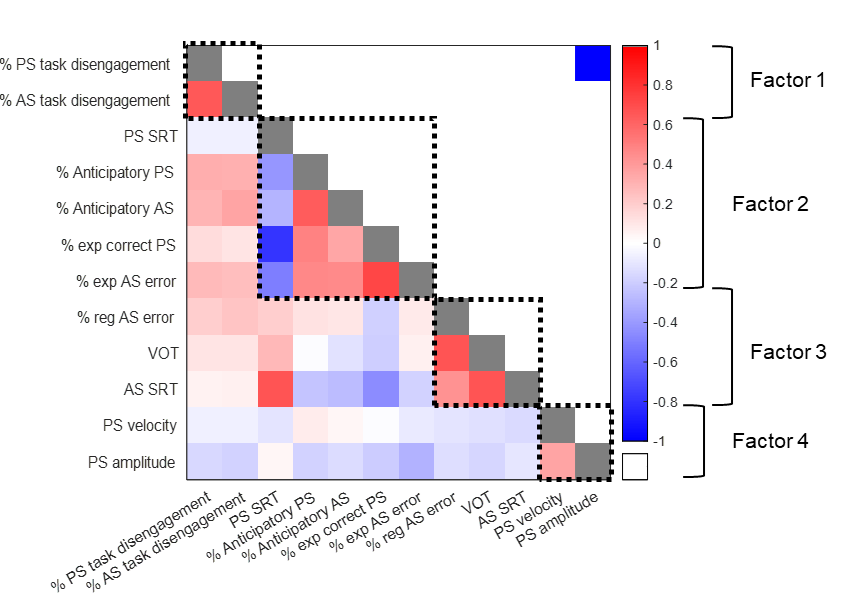

Supplement: Supplementary file 6 [file Image_5.TIF]
